# Inhibitory Effect of Morin Against *Candida albicans* Pathogenicity and Virulence Factor Production: An *in vitro* and *in vivo* Approaches

**DOI:** 10.3389/fmicb.2020.561298

**Published:** 2020-10-23

**Authors:** Gurusamy Abirami, Rajaiah Alexpandi, Ravindran Durgadevi, Arunachalam Kannappan, Arumugam Veera Ravi

**Affiliations:** ^1^Department of Biotechnology, School of Biological Sciences, Alagappa University, Karaikudi, India; ^2^Department of Food Science and Technology, School of Agriculture and Biology, Shanghai Jiao Tong University, Shanghai, China

**Keywords:** anti-biofilm, anti-virulence, anti-infective, morin, *Candida albicans*, zebrafish, systemic candidiasis

## Abstract

*Candida albicans* is considered an exclusive etiologic agent of candidiasis, a very common fungal infection in human. The expression of virulence factors contributes highly to the pathogenicity of *C. albicans*. These factors include biofilm formation, yeast-to-hyphal transition, adhesins, aspartyl proteases, and phospholipases secretion. Moreover, resistance development is a critical issue for the therapeutic failure of antifungal agents against systemic candidiasis. To circumvent resistance development, the present study investigated the virulence targeted therapeutic activity of the phyto-bioactive compound morin against *C. albicans*. Morin is a natural compound commonly found in medicinal plants and widely used in the pharmaceutical and cosmetic products/industries. The present study explicated the significant inhibitory potential of morin against biofilm formation and other virulence factors’ production, such as yeast-hyphal formation, phospholipase, and exopolymeric substances, in *C. albicans*. Further, qPCR analysis confirmed the downregulation of biofilm and virlence-related genes in *C. albicans* upon morin treatment, which is in correspondence with the *in vitro* bioassays. Further, the docking analysis revealed that morin shows strong affinity with Hwp-1 protein, which regulates the expression of biofilm and hyphal formation in *C. albicans* and, thereby, abolishes fungal pathogenicity. Moreover, the anti-infective potential of morin against *C. albicans*-associated systemic candidiasis is confirmed through an *in vivo* approach using biomedical model organism zebrafish (*Danio rerio*). The outcomes of the *in vivo* study demonstrate that the morin treatment effectively rescues animals from *C. albicans* infections and extends their survival rate by inhibiting the internal colonization of *C. albicans*. Histopathology analysis revealed extensive candidiasis-related pathognomonic changes in the gills, intestine, and kidney of animals infected with *C. albicans*, while no extensive abnormalities were observed in morin-treated animals. The results evidenced that morin has the ability to protect against the pathognomonic effect and histopathological lesions caused by *C. albicans* infection in zebrafish. Thus, the present study suggests that the utilization of morin could act as a potent therapeutic medication for *C. albicans* instigated candidiasis.

## Introduction

*Candida albicans* is an opportunistic fungal pathogen responsible for about 50–90% of all cases of candidiasis in humans. It is generally referred to as a dimorphic fungus since it grows both as yeast and filamentous cells (Romaní et al., 2006; Kadosh and Lopez-Ribot, 2013; Sardi et al., 2013; Salvatori et al., 2016; Tsui et al., 2016). The pathogenicity of *C. albicans* is aided by its exclusive virulence factors’ production such as biofilm formation, yeast-hyphal formation, and secretion of proteolytic and lipolytic enzymes (Galocha et al., 2019).

Among these virulent features, biofilm formation is imperious for *C. albicans* pathogenicity (Mohandas and Ballal, 2011). Biofilms of *C. albicans* are heterogeneous three-dimensional communities of yeast and hyphal cells within a self-secreted matrix of extracellular polymeric substances (EPS), which are comprised of polysaccharides, proteins, and nucleic acids (Lohse et al., 2018). *C. albicans* may form biofilms on the surface of implantable medical devices and has been involved in the establishment of device-associated nosocomial infections. The occurrence of invasive candidiasis in indwelling implant devices, such as catheters and stents, leads to persistent candidemia (Nucci, 2011). Moreover, biofilms have reduced susceptibility to antifungal drug therapy and host immune responses (da Costa et al., 2009).

Additionally, the surface-associated virulence factors, such as adhesins and invasins, have distinguishable roles in attachment and invasion into the host cells, which assists *C. albicans* in developing dexterous mechanisms to escape host immune responses (Mayer et al., 2013). Also, the degradative virulence enzymes, including phospholipases and proteases, strengthen the pathogenicity of *C. albicans* by degrading the cell membrane lipids and vital proteins of the skin (Rossoni et al., 2013).

Due to the imperative virulence factors production by *C. albicans*, it is very difficult to treat. Therefore, targeting such virulence factors could be considered as an effective alternative to prevent *C. albicans*-instigated infections. Recently, several plant-derived bioactive compounds have been explored for their antibiofilm activity against human pathogens (Shankar Raut and Mohan Karuppayil, 2016; Sivaranjani et al., 2016; Ramanathan et al., 2018). Hence, in the present study, morin was preferred to thwart *C. albicans* biofilm and other virulence factors’ production. Morin is a flavonoid found in several medicinal plants, including *Maclura tinctoria*, *Maclura pomifera*, and *Psidium guajava*, which exhibits a broad range of biological properties (Rattanachaikunsopon and Phumkhachorn, 2007). Several kinds of research have distinguished its anti-microbial, anti-inflammatory, anti-hypertensive, and anti-oxidant efficacy (Prahalathan et al., 2012; Abuohashish et al., 2013). In light of these facts, the present study was carried out to determine the antibiofilm and antivirulence activity of morin against *C. albicans* through *in vitro* bioassays and transcriptomic analysis. Further, *in vivo* analysis was performed to evaluate morin’s anti-infective potential against *C. albicans* instigated candidiasis in *Danio rerio* (Zebrafish) model system.

## Materials and Methods

### Fungal Strain and Culture Condition

The strain *C. albicans* ATCC 90028 was used in the present study. The test culture was maintained in Sabouraud dextrose agar (SDA; Himedia, Mumbai) and cultured routinely in YEPD (Yeast extract peptone dextrose) broth. For experimental analysis, a loopful of culture was used to inoculate YEPD medium and incubated at 37°C overnight. Prior to each bioassay, the overnight culture was adjusted to 0.8 OD at 600 nm (10^8^ CFU/ml). Spider broth (1% mannitol, 0.2% dipotassium hydrogen phosphate, and 1% nutrient broth) was used in the biofilm assay for augmenting hyphal formation (Muthamil and Pandian, 2016).

### Compound Preparation

Morin was obtained from Sigma-Aldrich (St. Louis, MO, United States). For experimental purpose, a stock solution of 50 mg/ml concentration of morin was prepared using DMSO.

### Effect of Morin on Growth of *C. albicans*

The antifungal effect of morin against *C. albicans* was assessed using the microbroth dilution method in a 24-well microtitre plate (MTP) as per the guidelines of the Clinical and Laboratory Standards Institute [CLSI] (2015). About 1% of overnight *C. albicans* culture was allowed to grow in 1 ml of YEPD broth in the presence and absence of morin (37.5–600 μg/ml). Wells holding 1 ml of plain YEPD broth served as a blank. The plate was incubated at 37°C for 16 h. After incubation, the growth absorbance was measured at OD_600 *nm*_ using a multi-mode plate reader (SpectraMaxM3, United States) (Baillie and Julia Douglas, 1998).

### Effect of Morin on Biofilm of *C. albicans*

The antibiofilm activity of morin was evaluated against *C. albicans* in a 24-well MTP (Ravindran et al., 2019; Alexpandi et al., 2020a). About 1% of test pathogen was added to 1 ml of spider broth along with morin at increasing concentrations, from 37.5, 75, to 150 μg/ml, and incubated at 37°C for 48 h. After incubation, the planktonic cells were discarded and the cells adhered in MTP wells were stained with 0.2% crystal violet. Then, the cells’ bound stains were eluted by 20% glacial acetic acid. The absorbance was measured at OD_570 *nm*_ using a multi-mode plate reader (SpectraMaxM3, United States) in order to evaluate the biofilm inhibition upon morin treatment. The least concentration of morin which showed more than 80% of biofilm inhibition was considered as the minimum biofilm inhibitory concentration (MBIC). The percentage of biofilm biomass inhibition was calculated by the following formula:

(1) $$\%\mathrm{of}\mathrm{inhibition}=[\left(\mathrm{Control}{\mathrm{OD}}_{570\text{nm}}-\mathrm{Treated}{\mathrm{OD}}_{570\text{nm}}\right)/\mathrm{Control}\mathrm{OD}]\times 100$$

### Effect of Morin on Exopolysaccharides Production of *C. albicans*

The effect of morin on exopolysaccharides production in *C. albicans* was quantified as described by Viszwapriya et al. (2016). The assay was performed with and without morin at increasing concentrations (37.5, 75, and 150 μg/ml) for 16 h. After incubation, both planktonic and biofilm cells were harvested by centrifugation at 12,000 rpm for 10 min and resuspended in 200 μl of 0.9% NaCl. An equal volume of 5% phenol was added to the cell suspension. Then, five volumes of H_2_SO_4_ was added to this reaction mixture and incubated in the dark at room temperature for 1 h. After incubation, the supernatant was collected and the absorbance was measured at OD_490 *nm*_.

### Microbial Adherence to Hydrocarbons Assay

The effect of morin on *C. albicans* cell surface hydrophobicity was assessed by microbial adhesion to hydrocarbons assay (Ravindran et al., 2018). The initial OD of morin treated and untreated *C. albicans* culture were taken at 600 nm. Then, equal volumes of toluene was added to the culture and vortexed vigorously for 10 min. The mixture was kept for phase separation. Then, the aqueous phase was separated and the absorbance was measured at OD_600 *nm*_. The percentage of hydrophobicity was calculated by the formula below:

(2) $$\%\mathrm{of}\mathrm{hydrophobicity}=[1-({\text{OD}}_{600\text{nm}}\mathrm{after}\mathrm{vortexing}/{\text{OD}}_{600\text{nm}}\mathrm{before}\mathrm{vortexing})]\times 100$$

### Microscopic Analysis

The antibiofilm activity of morin was visually evaluated using microscopic analyses as described by Kannappan et al. (2017) with minor modifications. *C. albicans* was allowed to form biofilm on the pieces of glass slides (1 cm × 1 cm) in the presence and absence of morin at MBIC. Then, both control and treated biofilm slides were stained with 0.2% crystal violet for light microscopy (Nikon Eclipse 80i, United States) analysis and 0.1% acridine orange for confocal laser scanning microscopy (CLSM) (Zeiss LSM 710, Germany) analysis. For scanning electron microscopic (SEM) analysis, biofilm cells were fixed in glass slides using 2.5% glutaraldehyde for 1 h at 4°C. Then, the fixed slides were gradually dehydrated with 20, 40, 60, 80, and 100% ethanol. Afterward, the slides were sputter coated with gold to observe under SEM (VEGA 3 TESCAN, Czech Republic).

### Growth Curve Analysis

The effect of morin on the cell proliferation of *C. albicans* was assessed by growth curve analysis. 1 ml of *C. albicans* culture (10^8^ CFU/ml) was used as the inoculum for 100 ml of YEPD broth, supplemented with and without morin (at MBIC), and incubated at 37°C. Then, the growth absorbance was measured at OD_600 *nm*_ for every 1 h of incubation period up to 16 h (Nithyanand and Pandian, 2009).

### Effect of Morin on *C. albicans* EPS Production

#### EPS Extraction

The cell-free and cell-bound EPS were collected from morin treated and untreated *C. albicans* culture, as described by Durgadevi et al. (2019). *C. albicans* cells were grown at 37°C for 16 h in YEPD media supplemented with and without morin. After incubation, the planktonic and biofilm cells of both morin-treated and -untreated samples were collected and centrifuged at 8,000 rpm for 10 min. The cell-free culture supernatant (CFCS) was stored at 4°C. Subsequently, the cell pellets were suspended in freshly prepared isotonic buffer (10 mM Tris, 2.5% NaCl, 10 mM EDTA) and incubated at 4°C overnight. After incubation, the cell-bound EPS containing supernatants were collected by centrifugation at 12,000 rpm for 15 min and pooled with the previously stored CFCS of respective control and morin treated *C. albicans* cultures. Then, 3 volume of ice-cold ethanol was added with each mixture and incubated at −20°C overnight. Finally, the precipitates of both cell-bound and cell-free EPS were collected by centrifugation at 12,000 rpm for 15 min.

#### Fourier Transform Infrared Spectroscopy Analysis

Fourier transform infrared (FT-IR) spectrophotometric (Nicolet iS5, Thermo Fisher Scientific Inc., United States) analysis was performed to observe changes in *C. albicans* EPS components upon morin treatment. For this, the extracted EPS samples were pelletized with potassium bromide at 1:100 ratio under hydraulic pressure. Then, the pelletized samples were scanned in the spectral range of 1800–500 cm^–1^. Then, the absorbance of both control and treated spectra was compared to observe the peak variations (Alexpandi et al., 2019).

### Inhibitory Effect of Morin Against Virulence Factor Production of *C. albicans*

#### Filamentous Morphology

The effect of morin on the filamentous morphology of *C. albicans* was evaluated as described by Subramenium et al. (2018). Spider agar plate was prepared with 1% Fetal bovine serum (FBS) in the presence and absence of morin at MBIC. Then, 3 μl of culture inoculum was spotted at the center of the agar plates and incubated at 37°C for 48 h. After incubation, the filamentous morphology of control and treated plates was documented using a high resolution charge-coupled device (CCD) camera (GelDoc XR+, Bio-Rad).

Further, the hyphal formation was enumerated using light microscopic analysis. About 1% of *C. albicans* was added to 1 ml of spider broth along with 1% FBS in the presence and absence of morin (at MBIC) at 37°C for 48 h. After incubation, 10 μl of the cells were placed on the glass slides and mounted using a cover slip. Then, three different spots in both the control and treated slides were randomly selected to be observed under light microscope.

#### Invasion

In order to assess the effect of morin on the adhesion of *C. albicans*, YEPD agar plates were prepared in the presence and absence of morin (at MBIC). The overnight culture was streaked on agar plates and incubated for 16 h at 37°C. After incubation, the plates were documented using a CCD camera (Romo et al., 2018).

#### Protease Production

The proteolytic activity of *C. albicans* was measured using azocasein as substrate. *C. albicans* was grown in 1 ml of YEPD broth in the presence and absence of morin and incubated at 37°C for16 h. After incubation, the CFCS were collected from each sample by centrifugation at 12000 rpm for 10 min. Then, 75 μl of CFCS was added to 125 μl of 2% azocasein and incubated at 37°C for 45 min. The reaction was stopped using 10% trichloroacetic acid and centrifuged at 12,000 rpm for 10 min. After centrifugation, the absorbance of the supernatants was measured at OD_440 *nm*_ (Alexpandi et al., 2019).

#### Phospholipase Production

For qualitative analysis of phospholipase production, 1 μl of inoculum was placed at the center of the SDA plate containing 1% peptone, 3% glucose, 5.73% NaCl, 0.055% CaCl_2_, and 10% of egg yolk emulsion in the absence and presence of morin (at MBIC), and the plates were then incubated at 37°C for 72 h (Ribeiro et al., 2010). The diameter of the precipitation zone around the colony was determined. Measurement and calculation of the zone of the phospholipase activity (*Pz*) were calculated as follows: colony diameter/ (colony diameter + precipitation zone). *Pz* coefficients were classified as negative (*Pz* = 1.00), positive (0.64 ≥ *Pz* < 1.00), and strongly positive (*Pz* < 0.64).

#### H_2_O_2_ Sensitivity Assay

To determine the effect of morin on *C. albicans* catalase production, a H_2_O_2_ sensitivity assay was performed (Sivaranjani et al., 2018). For this, 1% of *C. albicans* culture was swabbed over the SDA plates supplemented with and without morin (at MBIC). Then, sterile filter paper disks (Hi-Media, India) were placed at the center of the plates. About 20 μl of 30% H_2_O_2_ was loaded in the disks and the plates were incubated for 16 h at 37°C. After incubation, the diameter of the zones of clearance was measured. Subsequently, 100 μl of 30% H_2_O_2_ was added to morin treated and untreated *C. albicans* cell suspension and incubated at room temperature for 10 min. After incubation, the absorbance of each sample was measured at OD_240 *nm*_ in order to quantify the catalase activity (Hadwan, 2018).

### Eradication of Mature Biofilm and Light Microscopic Analysis

To determine the effect of morin on eradicating preformed biofilm, *C. albicans* was allowed to form biofilms in 24-well MTP containing 1 ml of spider broth supplemented with 1% inoculum at 37°C for 48 h. Then, the planktonic cells were removed and replaced with 1 ml of fresh spider broth supplemented with increasing concentrations of morin (37.5, 75, and 150 μg/ml) and incubated at 37°C for 16 h. After incubation, the non-adherent cells were washed with sterile PBS and allowed to dry. Then the biofilm biomass was quantified by the crystal violet staining method and visualized by light microscopic analysis (De Zoysa et al., 2018).

### Real-Time PCR Analysis

Total RNA was extracted from 16 h old *C. albicans* culture grown in the presence and absence of morin using TRIzol reagent, and quantification was done via BioSpec-nano spectrophotometer (Shimadzu, Japan). Isolated RNA was reverse transcribed into cDNA using a high capacity cDNA reverse transcription kit (Applied Biosystems). The primers were designed using primer3 software. The q-PCR was carried out using the SYBR Green PCR master mix in a 7500 Sequence Detection System (Applied Biosystems Inc., United States). The expression levels of the candidate genes (listed in Table 1) were normalized using housekeeping gene *ACT-1* (β-actin) of *C. albicans*. The relative gene expression level was determined by 2^–ΔΔ*Ct*^ calculation(Yadav et al., 2015).

**TABLE 1:** List of *C. albicans* virulence genes and their functions used in this study.

Gene	Function
*sap T1 sap T2*	Secreted aspartyl proteinases and involves in development and progression of candidiasis
*als1*	Agglutin-like sequences (promote biofilm formation).
*als2*	Role in adhesion and biofilm formation
*plb1*	Phospholipase (Secreted during invasion of the gastrointestinal tract)
*hwp1*	Hyphal wall protein (Cell surface protein and tight binding to oral epithelial cells).

### Molecular Docking

To identify the mode of the antibiofilm activity of morin, docking analysis was perfomed. The canonical SMILE of morin was downloaded from PubChem database and converted to a 3D-structure using an online smile translator server (Alexpandi et al., 2019). Subsequently, the protein sequence of Hyphal Wall Protein 1 (Hwp-1) was retrieved from Uniprot database [UniProt ID: Q14RS3] and its 3D-structure was generated using Phyre2 server. Then, molecular docking was performed using AutoDock Vina software and the ligand-protein interactions were visualized by Maestro 10 (Schrodinger) software (Balasubramaniam et al., 2019; Alexpandi et al., 2020b).

### *In vivo* Studies With Zebrafish Model

#### Animal Maintenance

The wild type adult zebrafish (*Danio rerio*) were obtained from an aquaculture farm (Sridhar Aquarium, Chennai, India). The collected zebrafish were acclimatized at 28 ± 2°C for seven days in a glass aquarium containing filtered fresh water and fed with commercial food pellets twice a day. All the *in vivo* experiments were performed in accordance with the guidelines of the Committee for the Purpose of Control and Supervision of Experiments on Animals(CPCSEA) (cpcsea.nic. in/WriteReadData/userfiles/file/SOP_CPCSEA_inner_page.pdf), Government of India, and the general guidelines of the Institutional Animal Ethics Committee, Alagappa University (Reg No: 183 2007/GO/ReBi/S/18/CPCSEA dt 14.03.2018). The healthy and equal-sized fishes were selected for the *in vivo* experiments. All *in vivo* experiments were performed in triplicate.

#### Toxicity Assessment

The toxicity test was carried out to determine the non-lethal concentrations of morin. For this, the healthy acclimatized fish were divided into five groups – one for normal control and four for morin at different concentrations (37.5, 75, 150, and 300 μg/ml) – in a fresh water (1 L) aquarium. Each group consisted of six animals and the survival percentage was monitored for every 12 h up to 96 h of the post-compound treatments. The concentration at which an 100% survival rate of fish occurred after 96 h was considered as the non-lethal concentration (LD_0_). All further experiments were made only at non-lethal concentrations.

### Infection Study

For revealing the anti-infective potential of morin against *C. albicans* infection, the healthy zebrafish were infected with *C. albicans* (10^8^ CFU/ml) under aeration in a plastic pocket for 12 h. Then, the post-infected animals were transferred into an aquarium containing either normal or morin (37.5 and 75 μg/ml) treated water (1 L). The survival rate of infection control (post-infected fish) and treated (post-infected fish + morin) groups was assessed every 12 h up to 96 h.

### Effect of Morin on Internal Colonization of *C. albicans* in Zebrafish

After the infection study, *C. albicans*-infected control and treated animals were sacrificed and homogenized in 1 ml of PBS. Then, the slurry was serially diluted and spread plate on the SDA medium and incubated at 37°C for 24 h. After incubation, the CFU of each sample was counted.

### Histopathology Analysis

To analyze the systemic infections caused by candidiasis in zebrafish, the infected control and treated zebrafishes were fixed with 10% (v/v) phosphate-buffered formalin containing 0.4% NaH_2_PO_4_, 0.65% Na_2_HPO_4_, and 40% formalin. Then, the animals were preserved in 70% ethanol until processing. Subsequently, the samples were dehydrated and embedded in paraffin wax. The tissues were sectioned transversely and stained with hematoxylin and eosin. The samples were examined using a light microscope and documented in Nikon Eclipse, Ti 100.

### Statistics

All the experiments were performed at least thrice independently. The statistical significance of data was analyzed by Student’s *T*-test using SPSS statistics v17.0 software package to study the statistical difference between control and treated samples. The significant value was fixed at *p* ≤ 0.05.

## Results

### Antibiofilm and Non-fatal Effect of Morin Against *C. albicans*

**FIGURE 1:**
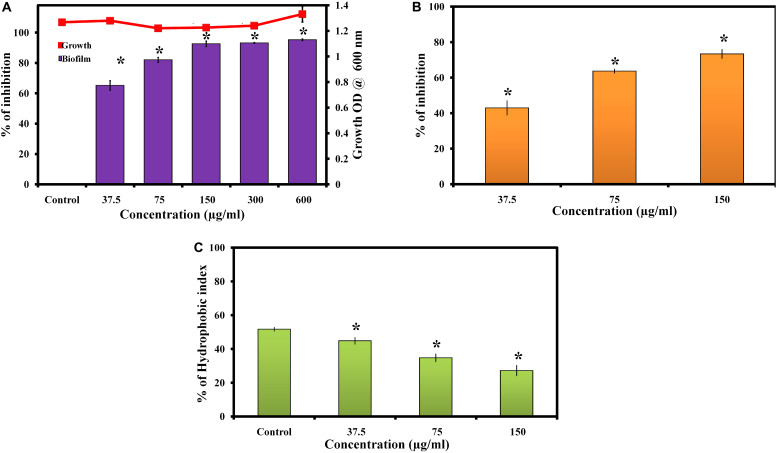
**(A)** Effect of morin on growth and biofilm of *C. albicans*. Data are presented as means ± SD. *Indicates statistical significance (*p* < 0.05). **(B)** Inhibitory effect of morin on *C. albicans* exopolysaccharide production. Data are presented as means ± SD. *Indicates the statistical significance (*p* < 0.05). **(C)** Effect of morin on cell surface hydrophobicity of *C. albicans*. Data are presented as means ± SD. *Indicates statistical significance (*p* < 0.05).

Morin exhibited concentration dependent biofilm inhibition in *C. albicans*. At 150 μg/ml, morin displayed a maximum of 93% biofilm inhibition. Hence, 150 μg/ml of morin was considered as MBIC and used for all *in vitro* assays. Also, the effect of morin on *C. albicans* growth was observed by microbroth dilution method, which showed the non-antifungal nature of morin up to 600 μg/ml concentration (Figure 1A).

### Effect of Morin on Exopolysaccharides Production of *C. albicans*

The effect of morin on exopolysaccharides production was measured by quantifying the total carbohydrate using the phenol sulphuric acid method. Noteworthily, morin efficiently inhibited EPS production in a dose-dependent manner (Figure 1B); the results revealed significant inhibition of exopolysaccharides at 150 μg/ml concentration to the level of 73%.

### Effect of Morin on Hydrophobicity Formation of *C. albicans*

Cell surface hydrophobicity has a major influence on microbial adhesion by enhancing hydrophobic interactions between microbial cells and biological or nonbiological surfaces. The effect of morin on the hydrophobicity of *C. albicans* was determined by MATH assay. In MATH assay, the hydrophobicity index of morin treated *C. albicans* was 45, 35, and 27% at 37.5, 75, and 150 μg/ml concentrations, respectively, and the hydrophobicity index of control was 52% (Figure 1C). As expected, the morin effectively reduced the hydrophobicity of *C. albicans* compared to the untreated control.

### *In-situ* Visualization of Biofilm Inhibition

**FIGURE 2:**
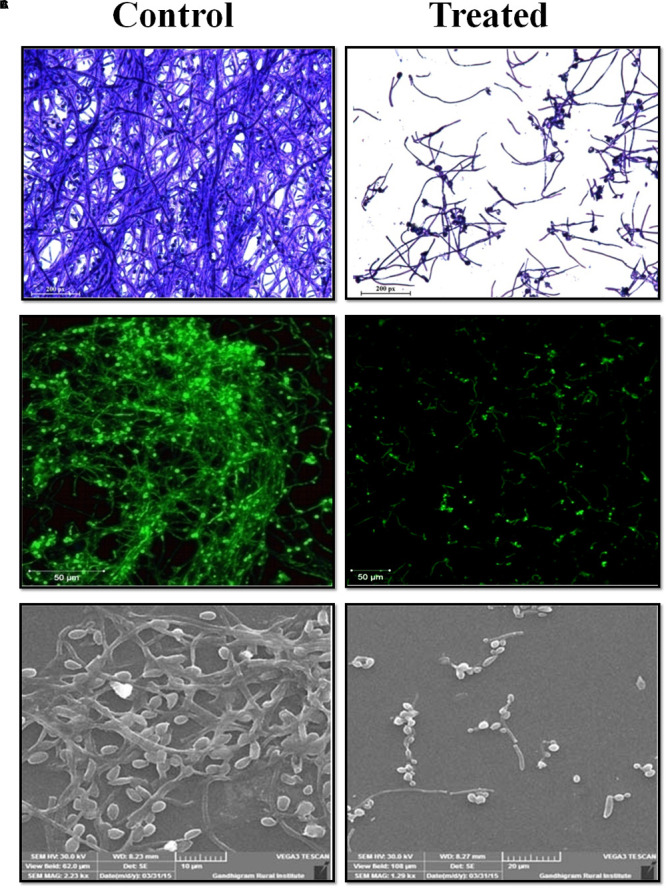
Microscopic analysis of *C. albicans* biofilms in the absence and presence of morin at MBIC. **(A)** Light microscope. **(B)** Confocal laser scanning microscope. **(C)** Scaning electron microscope analysis.

The antibiofilm potential of morin against *C. albicans* was visualized by *in situ* microscopic analyses. In Figure 2A, light microscopic and CLSM images of morin-treated (at MBIC) samples depict a highly reduced biofilm architecture, while the untreated control images show a dense and multilayered biofilm matrix. Further, SEM analysis of *C. albicans* biofilm clearly depicts a robust and highly structured biofilm matrix in the untreated control sample whereas the treated sample showed effectually reduced biofilm-associated *C. albicans* cells (Figures 2B,C).

### Effect of Morin on Growth Pattern of *C. albicans*

**FIGURE 3:**
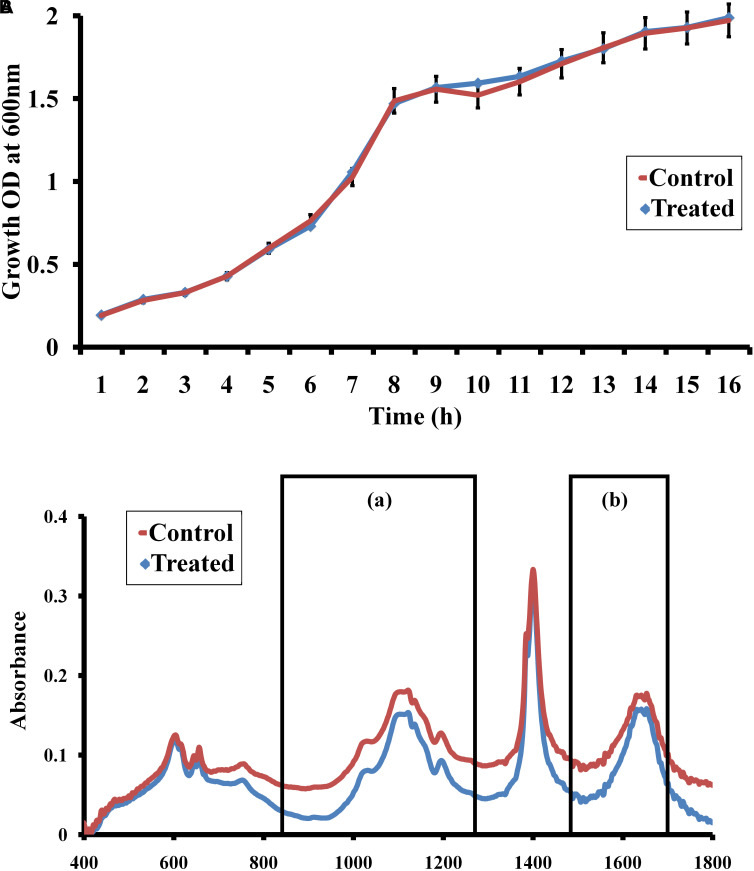
**(A)** Growth curve analysis. The graph represents the effect of morin (at MBIC) on the growth dynamics of *C. albicans*. **(B)** FTIR spectra of EPS samples extracted from morin-treated (at MBIC) and untreated *C. albicans*. FT-IR spectra showed variations in regions such as **(a)** mixed regions of polysaccharides and nucleic acids (1300-900 cm^–1^). **(b)** proteins (1700–1500 cm^–1^),

Growth curve analysis was performed to confirm the non-fatal effect of morin (at MBIC) in *C. albicans*. The result of the growth curve analysis indicates that the growth pattern of morin-treated (at MBIC) *C. albicans* was similar to that of the untreated control (Figure 3A).

### FT-IR Analysis

Fourier transform infrared analysis was performed to analyse the EPS inhibitory activity of morin, wherein the IR spectra of control and treated samples were comparatively analyzed. Herein, the intensity of peak absorbance of the morin-treated sample at 1700–1500 cm^–1^ and 1300–900 cm^–1^. representing the proteins and mixed regions of polysaccharides and nucleic acids, respectively, was highly reduced when compared with the intensity of the control spectra (Figure 3B).

### Inhibitory Effect of Morin Against *C. albicans* Virulence Factor Production

#### Effect on Filamentous Morphogenesis

**FIGURE 4:**
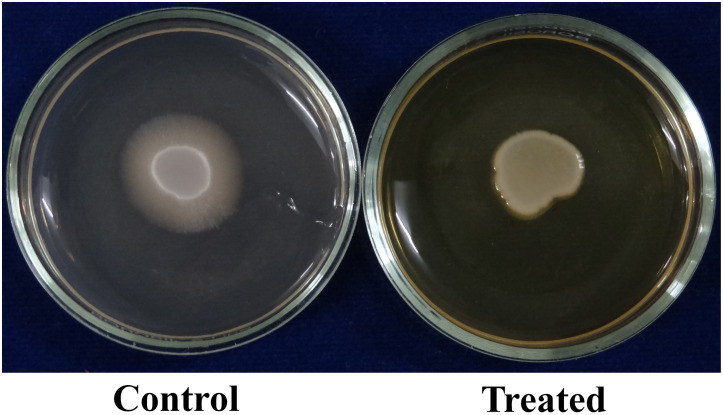
Effect of morin on filamentous morphology. Hyphal morphology was significantly reduced at MBIC concentration (at MBIC).

Hyphal production is an imperious virulence factor in *C. albicans* pathogenesis. Transformation of yeast cells to hyphal cells promotes the invasion and adhesion of pathogenic *C. albicans*. In this study, the hyphal morphology was significantly reduced in the morin-treated spider agar plate when compared to the untreated control (Figure 4). Further, light microscopic analysis was performed to quantify the filamentous growth in morin-treated and untreated control. The three randomnly selected spots showed that the untreated control sample has a highly structured hyphal growth. Conversely, the morin-treated (at MBIC) sample showed a small and reduced number of hyphal formation. So, the single spot of filamentous growth in the control and treated slide were represented. Then, the number of cells were enumerated manually from three different spots of untreated control and morin-treated, which is proof of the inhibitory potential of morin against filamentous morphogenesis of pathogenic *C. albicans* (Supplementary Figures 1A,B).

#### Effect on Invasion

**FIGURE 5:**
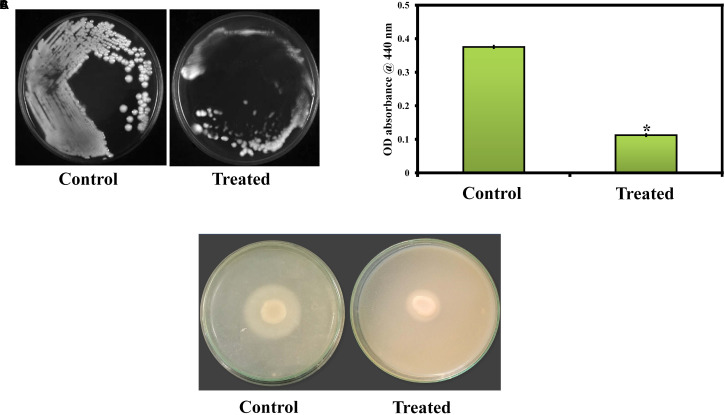
**(A)** Activity of morin against *C. albicans* in an agar invasion assay. **(B)** Effect of morin on *C. albicans* protease production at the increasing concentration (37.5, 75, and 150μg/ml). Data are presented as means ± SD. *Indicates the statistical significance (*p* < 0.05). **(C)** Effect of morin (at MBIC) on *C. albicans* phospholipase production.

*C. albicans* can invade the host epithelial cells by endocytosis. Following the invasion of epithelial cells, *C. albicans* can penetrate more deeply into blood vessels and invade into the bloodstream. In the present study, the morin-(MBIC) treated agar plate shows loosely attached *C. albicans* colonies, whereas the robust colonies of *C. albicans* were observed in the untreated agar plate (Figure 5A).

#### Effect on Protease Production

In the present study, the protease production in *C. albicans* was quantified by azocasein assay. Morin exhibits significant inhibitory activity against *C. albicans* protease production at its MBIC compared to that of the untreated control (Figure 5B).

#### Effect on Phospholipase Production

The phospholipase production in *C. albicans* was qualitatively as well as quantitatively estimated in SDA agar plates containing appropriate substrates in the absence and presence of morin at MBIC. In Figure 5C, the zone of precipitation was found to be reduced around the untreated fungal colony when compared to the treated colony. Further, the phospholipase activity (*Pz* value) of the control and treated samples was found to be 0.31 and 0.42, respectively. These *Pz* values (*Pz* < 0.64) represent the strongly positive phospholipase activity of *C. albicans*, as mentioned above. Also, the low *Pz* values mean high phospholipase production and, conversely, high *Pz* values indicate low enzymatic production (Ishida et al., 2012). Hence, the observed *Pz* values of control and treated samples revealed the inhibition of phospholipase production in *C. albicans* upon morin (at MBIC) treatment.

#### Effect on H_2_O_2_ Sensitivity

**FIGURE 6:**
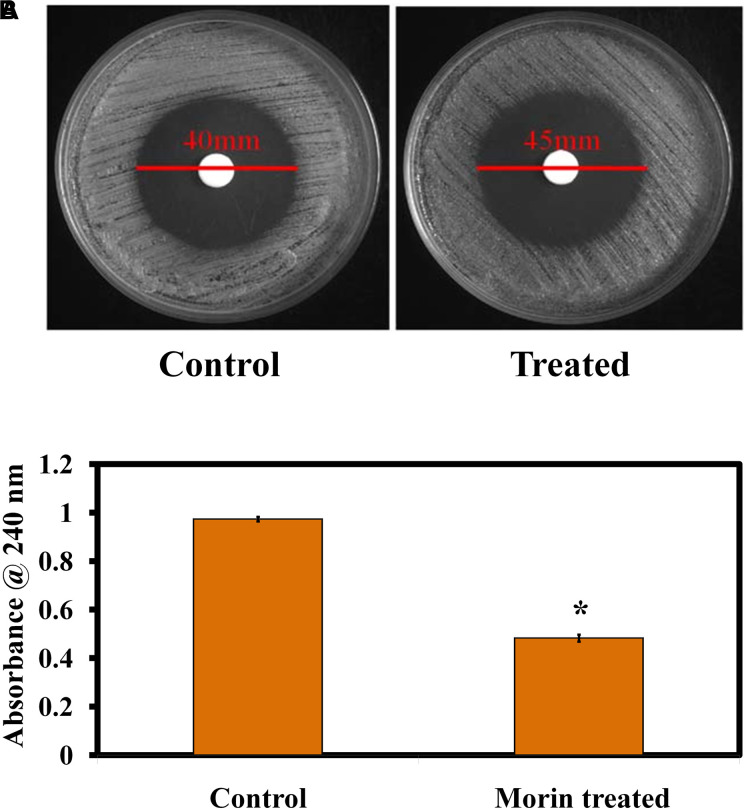
**(A)** Effect of morin (at MBIC) on *C. albicans* catalase production. **(B)** Quantitative analysis of the catalase inhibitory activity of morin (at MBIC).

The effect of morin treatment on catalase production in *C. albicans* was evaluated by the H_2_O_2_ disk diffusion assay. The obtained results illustrate that the H_2_O_2_ sensitivity of morin-treated *C. albicans* was higher than that of the untreated control. The diameter of the H_2_O_2_ sensitivity zone of inhibition in the morin-treated plate was found to be 45 mm whereas the diameter of zone of inhibition in the untreated *C. albicans* plate was 40 mm (Figure 6A). Further, the H_2_O_2_ tube assay was perfomed to quantify the catalase production. In Figure 6B, morin exhibits significant inhibitory activity against *C. albicans* catalase production at its MBIC compared to that of the untreated control.

#### Effect on Mature Biofilms

The preformed biofilm disruption efficacy of morin against *C. albicans’* biofilm was assessed using the crystal violet staining. The results clearly revealed that a maximum 55% of preformed biofilm was destroyed upon morin-(at MBIC) treatment (Supplementary Figure 2A). Furthermore, the light microscopic analysis validated the preformed biofilm eradication efficacy of morin, in which the morin- (at MBIC) treatment prominently disturbed the 48 h mature biofilm, compared to the untreated control slide (Supplementary Figure 2B).

#### Effect of Morin on Virulence Gene Expression in *C. albicans*

**FIGURE 7:**
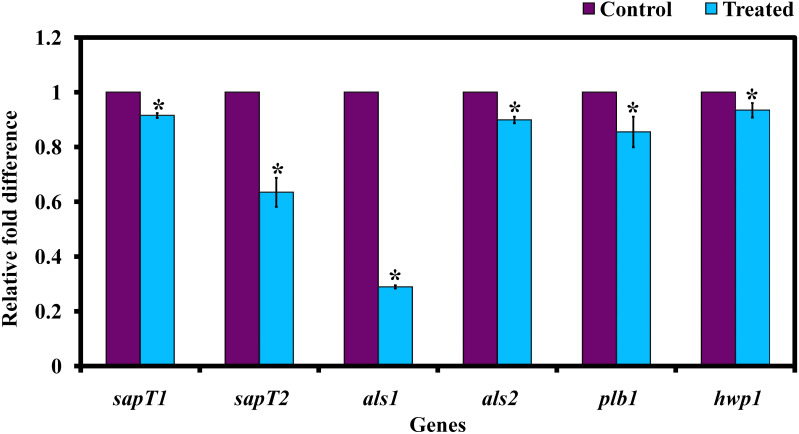
Gene expression analysis of *C. albicans* in the presence and absence of morin (at MBIC). Data are presented as means ± SD. *Indicates statistical significance (*p* < 0.05).

The relative expression level of *C. albican’s* virulence traits was assessed by real-time PCR (qPCR) analysis. The obtained result exhibited that the genes *sapT1*, *sapT2*, *als1*, *als2*, *plb1*, and *hwp1* are significantly downregulated upon morin-(at MBIC) treatment (Figure 7).

#### Interaction Analysis of Morin With Hwp1 Receptor

**FIGURE 8:**
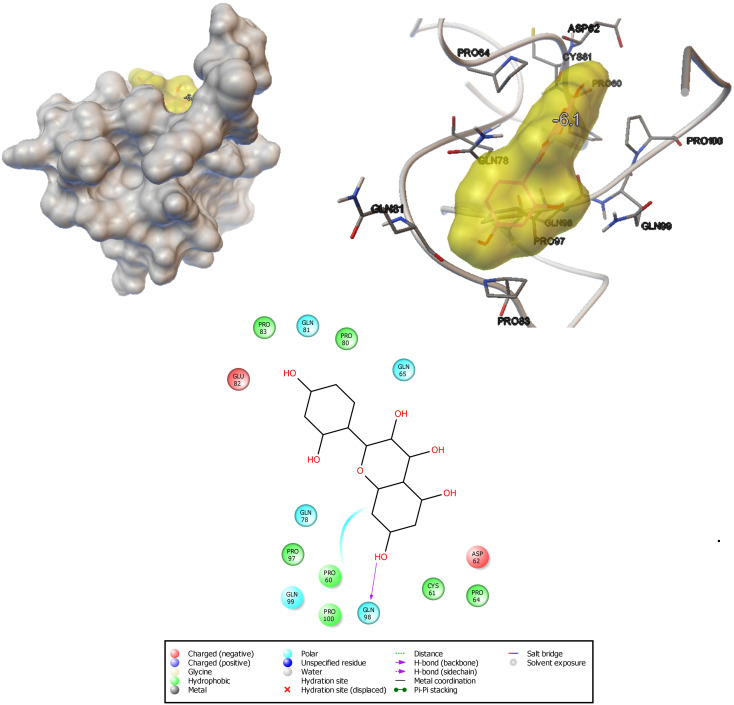
Docking analysis shows the binding pattern and amino acid interactions of morin (−6.1 kcal/mol) with Hwp1 receptor in *C. albicans* for possible inhibition of adhesion as well as biofilm formation.

Using an *in silico* molecular docking approach, the potential of morin to interact with *C. albicans* hyphal wall protein (Hwp1) was measured. The results of molecular docking analysis revealed that the ligand molecule (morin) (6.1 Kcal/mol) builds seven hydrophobic interactions (Pro83, Pro80, Pro97, Pro60, Cys61, and Pro64), five polar interactions (Gln81, Gln65, Gln78, Gln98, and Gln99), and two negative-charge interactions (Glu82 and Asp62) within the region of the receptor (Figure 8). The ligand molecule especially strongly interacts with Gln98 via hydrogen bonding. This observation suggests that the strong interactions between the ligand and Hwp1 receptor molecule can potentially lead to the inhibition of the hyphal adhesion in *C. albicans*.

### *In vivo* Study Using Zebrafish Model

#### Toxicity Assessment of Morin

**FIGURE 9:**
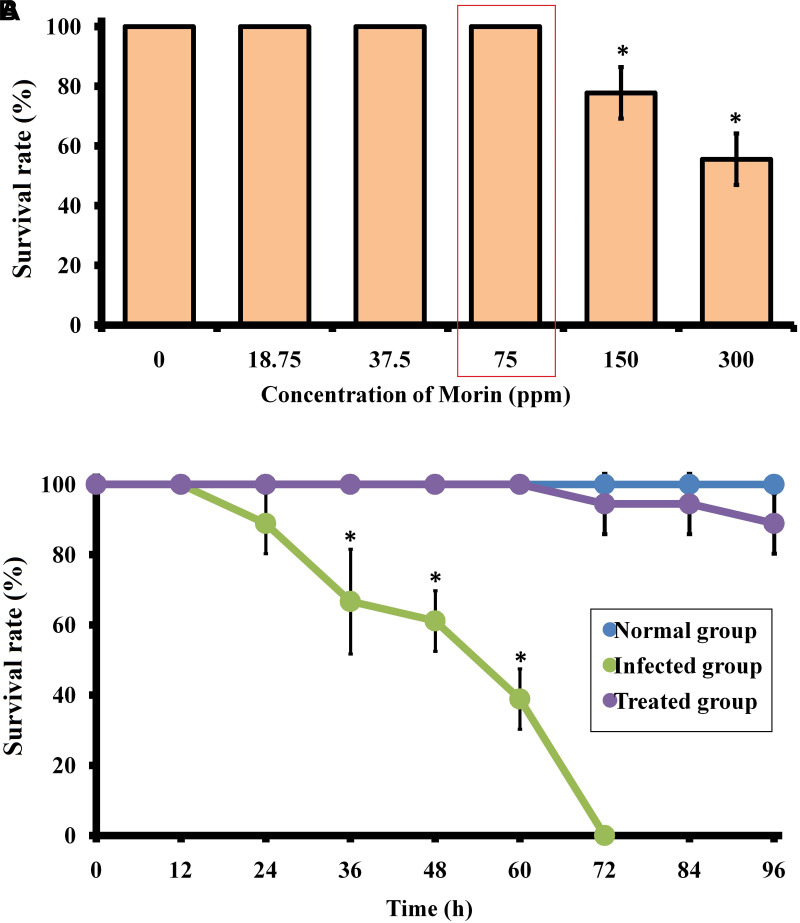
**(A)** Surival percentage of zebrafish in the presence of morin at different concentrations. **(B)** Survival percentage of *C. albicans*-infected zebrafish upon morin treatment (75 μg/ml) up to 96 h. Data are presented as means ± SD. *Indicates statistical significance (*p* < 0.05).

In the present study, the LC_50_ and LD_0_ values of morin to zebrafish were found to be ∼300 and 75 μg/ml, respectively. The non-lethal concentration of 75 μg/ml was used for further studies (Figure 9A).

#### Zebrafish Survival Assay

The therapeutic efficacy of morin on *C. albicans*-infected zebrafish was tested over a 96 h time period. The obtained results showed an 100% mortality in the post-infected group of zebrafish at 70 h, whereas an 88.8% survival rate was maintained in the morin- (75 μg/ml) treated post-infected group of zebrafish up to 96 h (Figure 9B). Also, a better survival rate was observed in the treated post-infected group compared to their infected controls which strongly suggest the *in vivo* disinfection activity of morin against *C. albicans* infections (Supplementary Figure 3). This observation reveals that morin-treated animals recovered from the infections caused by *C. albicans*

#### Evaluation of *C. albicans* Load in Zebrafish

The effect of morin treatment on the internal colonization level of *C. albicans* infected zebrafish was assessed by spread plate method. The observed results shows a significantly reduced level of colony counts in morin-treated post-infected animal samples when compared to the infected control. The CFU level of the treated group was observed as 1.47 × 10^4^, whereas 1.63 × 10^5^ CFU was observed in the infected control sample (Supplementary Figure 4).

#### Histopathology Analysis

**FIGURE 10:**
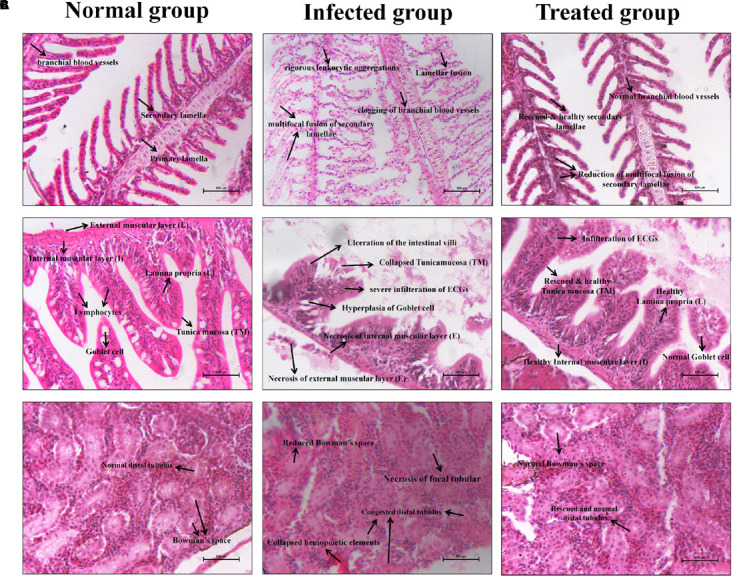
Histopathology analysis of the Gills **(A)**, Intestine **(B),** and Kidney **(C)** sections of uninfected control, *C. albicans* infected control and morin treated zebrafishes.

The infected control and treated zebrafishes’ gills, intestine, and kidney samples were collected for histopathological analysis. In Figure 10A, the gills’ histopathology of infected and treated groups shows the regular and usual structures without any significant lesions in the uninfected control sample. Whereas, the lamellar fusion and multifocal fusion of the secondary gill lamellae due to the hyperplasia of the epithelium of the primary lamellae were observed in the *C. albicans*-infected sample. Also, clogging of branchial blood vessels with a multifocal blend of secondary lamellae and rigorous leukocytic aggregations at the primary gill lamellae were observed in the infected group, which symbolizes the respiratory infective effect of *C. albicans*. However, these lesions of filamentous clubbing, mucous cells hyperplasia, lamellar telangiectasis, and entire fusion of secondary lamellae were not observed in the morin-treated sample, which indicates the rescue action of morin against *C. albicans* infection.

In Figure 10B, the intestine histopathology of the uninfected control group showed normal intestinal villi and usual tunica mucosa (TM), Lamina propria (L), Internal muscular layer (I), External muscular layer (E), and Goblet cells without any significant pathological changes. In contrast, the intestine of the infected group displayed focal necrosis of the intestinal epithelium of tunica mucosa and lamina propria with severe infiltration of submucosal eosinophilic granular cells (EGCs) and hyperplasia of the goblet cells. The disgusting abnormalities of enlargement of the epithelium, membrane damage, and ulceration of the intestinal villi were observed after *C. albicans* infection, which are represented by the lesions of gastrointestinal candidiasis in zebrafish. Interestingly, these deleterious effects were protected in the treated group. But, some membrane scratches of intestinal villi and infiltration of EGCs were observed, without perilous effects.

In Figure 10C, the kidney histopathology of the uninfected control group shows no histopathologic changes. The kidney of the infected group displays congested distal tubules, focal tubular necrosis, collapsed hemopoietic elements, and also a prominently reduced Bowman’s space. Interestingly, these deleterious defects were significantly rescued in the treated group upon morin treatment.

## Discussion

*Candida albicans* is the most common human fungal pathogen, causing diseases ranging from mucosal to systemic infections. The majority of *C. albicans* infections are associated with its ability to form biofilms (Nett and Andes, 2006). Moreover, cells are enclosed in recalcitrant biofilms which cause resistant host defense mechanisms and impair the effectual action of conventional antifungals, therefore leading to a higher chance of multidrug resistance development (Tournu and Van Dijck, 2012; Balaji et al., 2020; Durgadevi et al., 2020). Therefore, there is a need to develop an alternative treatment strategy to combat biofilm-associated *Candida* infections. To cope with this issue, this study was intended to explore the antibiofilm and antivirulence activity of morin against *C. albicans*.

To begin with, the antibiofilm activity of morin was analyzed by biofilm biomass quantification method. In this study, the MBIC of morin was found to be 150 μg/ml at which a maximum of 93% inhibition with no adverse effects on *C. albicans* growth was observed. Also, the non-fatal antibiofilm potency of morin was confirmed through growth curve analysis, wherein morin treatment (at MBIC) did not show any significant lethal effect on the growth pattern of *C. albicans*. In addition, the mature (48 h) biofilm of *C. albicans* was efficiently degraded by morin treatment at its MBIC and the light microscopic analysis further validated this observation. The outcome of the present study supports the previous study of Subramenium et al. (2018), which reported the efficiency of marine bacterial extracts to inhibit the biofilm formation of *C. albicans* by reducing EPS production. Since EPS plays a key role in construction of the biofilm matrix (Bujdáková et al., 2013; Ravindran et al., 2019), the efficacy of morin treatment on *C. albicans* EPS production was assessed in the present study. From the FT-IR analysis, the potential EPS inhibitory activity of morin (at MBIC) against *C. albicans* was confirmed. These observations conclude that morin treatment efficiently inhibits *C. albicans* biofilm production by interfering with EPS production. The hydrophobicity of microbial pathogens contributes to their adherence, which is responsible for biofilm formation and adhesion to epithelial cells. The adherence of *C. albicans* to the epithelial surface is considered to be an important factor in the development of infection (Cotter and Kavanagh, 2000). In the present study, morin treatment significantly reduced the hydrophobicity index of *C. albicans* when compared to the untreated control.

The yeast-to-hyphal transition is believed to be a vital factor for the pathogenicity of *C. albicans*. The hyphal cells penetrate endothelial and epithelial tissues for colonization and invade the yeast cells into the bloodstream (Azadmanesh et al., 2017). This led us to investigate the effect of morin on yeast-to-hyphal transition by filamentation assay. The result revealed the reduced hyphal formation in morintreated cells and a high filamentous form of control cells. The microscopic analyses of *C. albicans* cells also unveil the highly reduced filamentous growth in morin-treated cells and robust filamentous growth of control cells. In addition, the *in silico* docking analysis validated the antihyphal activity of morin by demonstrating the strong affinity of the ligand morin with the key regulator hyphae (Hwp1) protein. Hyphal wall protein (Hwp1) is highly expressive in *C. albicans* yeast – hyphal transition (Nobile et al., 2006). Thus, the present study affirmed the potential of morin in impeding the major virulence of *C. albicans* aiding invasion.

Apart from morphological transition, the secreted virulence factors of *C. albicans*, such as proteases and phospholipases, contribute to elevating its pathogenicity. These hydrolases proficiently degrade host membrane and surface membrane proteins, thereby aiding in the invasion of *C. albicans* into host tissue (Mayer et al., 2013). Morin was effective in impeding such virulence enzymes production of *C. albicans*. This observation led us to envisage that morin treatment might be able to deter the development of candidemia. In addition, the effect of morin on the production of antioxidant enzyme catalase by *C. albicans* was assessed, as aerobic organisms secrete oxygen by-products (H_2_O_2_), known as reactive oxygen species. Morin was effective in impeding catalase production of *C. albicans*, which was confirmed through a H_2_O_2_ sensitive test and tube assay.

Further, the expression level of virulence genes upon morin treatment was evaluated for validating the *in vitro* bioassay results. In *C. albicans*, agglutinin-like sequence (ALS) genes, including ALS1 and ALS2, play a crucial role in adhesion-mediated biofilm formation by *C. albicans* to cause candidiasis. Also, ALS and HWP1 are simultaneously expressed in *C. albicans* hyphal cells. In the present study, the qPCR analysis reveals that morin treatment significantly downregulated the expression level of *als1*, *als2*, and *hwp1* genes, which correlated well with the observation of the *in vitro* filamentation assay. And the genes *sap* and *plb* are also responsible for adhesion and biofilm formation by *C. albicans*, which is also involved in the secretion of extracellular hydrolytic enzymes such as protease, esterase, and lipase (Naglik et al., 2003). The gene expression analysis revealed that the expression level of *SapT1*, *SapT2*, and *plb1* genes were significantly downregulated upon morin treatment.

For studying the anti-infective potential of morin against fungal infections, zebrafish are more robust and advantageous than other model organisms (Gomes and Mostowy, 2020). Since the genome of zebrafish was 80 % conserved in the human genome, zebrafish are reported as a perfect model host for monitoring the progression of *C. albicans* colonization, invasion in multiple organ sites, host-pathogen interactions, and pathogenicity (Rosowski et al., 2018). In the present study, all the *in vitro* results demonstrated that morin is a suitable therapeutic agent for dealing with invasive fungal infections. As it is an important criterion for a compound to be non-toxic for clinical applications, the toxicity of morin was assessed on an *in vivo* model organism zebrafish. The results of the toxixity study revealed that the survival rate of the morin- (75 μg/ml) exposed animal group was similar to that of the untreated control group, which portrays the non-lethal effect of morin. Also, the survivability of *C. albicans*-infected animals were completely rescued upon morin (75 μg/ml) treatment, which is accomplished by hampering the internal colonization of *C. albicans*. Since internal accumulation of *C. albicans* results in biofilm formation in the mouth, throat, vagina, and other internal organs, including the kidney, intestinal, spleen, heart, and brain, it is highly related to the pathogenesis of candidiasis (Kwun and Lee, 2020). The CFU assay exhibited that the fungal count in morin-treated post-infected animal samples was significantly reduced when compared to the untreated animals, which unveiled the anti-infective potential of morin against *C. albicans* infections.

Furthermore, the histopathological observations attested to the systemic infection of *C. albicans* through perceiving respective pathologic lesions in the tested organs, including the gills, intestine, and kidney (Ahmed et al., 2016). Gills are the main respiratory organs in zebrafish and hence was the first site for the colonization of microbial pathogens (Griffitt et al., 2009). In this manner, it is more defenseless against harm than other tissues. Similarly, the obtained results established that the *C. albicans*-infected group shows hyperplasia of the epithelium, lamellar fusion and multifocal fusion of the secondary gill lamellae, clogging of branchial blood vessels, and rigorous leukocytic aggregations at the primary gill lamellae (Chao et al., 2010), which symbolizes the respiratory candidiasis effect of *C. albicans*. As expected, the absence of these lesions in the treated group indicated the rescue action of morin treatment against *C. albicans* infection. Subsequently, the gross abnormalities, such as focal necrosis of tunica mucosa and lamina propria, severe EGCs infiltration, ulceration of the intestinal villi, and hyperplasia of the goblet cells, are denoted in the intestinal candidiasis in the infected group (Gonia et al., 2017), which are also oberved in *C. albicans* infected test organs’ histopathology images. The disgusting abnormalities of congested distal tubulus, focal tubular necrosis, collapsed hemopoietic elements, and also the Bowman’s space, is associated with the UTI-related pathogenic effect in the kidney of infected group (Rodríguez et al., 2009; Chang et al., 2016). However, the successful recovery of these lesions in the intestine and kidney of the treated group strongly proved the *in vivo* disease protection efficacy. Overall, the findings of the current study demonstrate that morin is a promising antipathogenic agent against *C. albicans* and holds great potential to be used as a therapeutic agent to combat *C. albicans* mediated systemic candidiasis.

## Conclusion

Based on the present study, it could be concluded that morin treatment significantly inhibited the biofilm formation of *C. albicans* in a concentration-dependent manner. Further, the production of virulence factors, such as hyphal formation, phospholipase, protease, invasion, and exopolysaccharides, were also significantly abridged upon the morin treatment at its MBIC. In addition, gene expression analysis clearly revealed that the expression level of biofilm and virulence-associated genes were significantly downregulated upon morin treatment. Also, docking analysis revealed the inhibitory potential of morin against biofilm formation and hyphal production by analysis of the interaction ability of morin with Hwp1 receptor protein in *C. albicans*. In addition, the *in vivo* analysis revealed the anti-infective potential of morin by protecting the animals from the pathogenicity of *C. albicans*. Overall, the present study reports the feasibility of morin acting as a promising therapeutic agent against *C. albicans*-mediated systemic candidiasis.

## Data Availability Statement

All datasets presented in this study are included in the article/Supplementary Material.

## Ethics Statement

Ethical review and approval was not required for the animal study because all the experiments in zebrafish were performed followed by the guidelines agreed by the Committee for the Purpose of Control and Supervision of Experiments on Animals (CPCSEA), Government of India (cpcsea.nic. in/WriteReadData/userfiles/file/SOP_CPCSEA_inner_page.pdf). Hence, this is not mandatory regarding the ethical issues, as it is not mentioned in the CPCSEA guideline yet.

## Author Contributions

GA: conceptualization, performed the experiments, data analysis, and writing – original draft. RA: performed the *in vivo* experiments, data analysis, and reviewing original draft. RD and AV: data analysis and reviewing original draft. AV: conceptualization, supervision, data validation, and writing – original draft. All authors contributed to the article and approved the submitted version.

## Conflict of Interest

The authors declare that the research was conducted in the absence of any commercial or financial relationships that could be construed as a potential conflict of interest.

## Supplementary Material

Supplementary Figure 1 **(A)** Enumeration of filamentous cells in the presence and absence of morin at its MBIC. Data are presented as means ± SD. ^∗^ indicates the statistical significance (p < 0.05). **(B)** Light microscopic analysis of single spot filamentous cells in the presence and absence of morin at its MBIC were represented.

Supplementary Figure 2 **(A)** Inhibitory effect of morin on *C. albicans* mature biofilm production. **(B)** Light microscopic images showing the effective non-inhibitory activity of morin at MBIC on preformed biofilm of *C. albicans*. Data are presented as means ± SD. ^∗^ indicates statistical significance (p < 0.05).

Supplementary Figure 3 Photographs illustates the clinical symptoms of *C. albicans* infection in zebrafish in the presence and absence of morin treatment. In *C. albicans* infected group, the black arrows represent the hemorrhaging at visceral organs of zebrafish such as (a) gills (b) gastrointestinal tract (c) urinary tract upon *C. albicans* infection.

Supplementary Figure 4 Effect of morin on *in vivo* colonization of *C. albicans* in zebrafish. The reduction number of *C. albicans* colonization on zebrafish was assessed by CFU assay.
