# Supplementary material for: Inhibitory Effect of Morin Against Candida albicans Pathogenicity and Virulence Factor Production: An in vitro and in vivo Approaches
Source: Front Microbiol. 2020 Oct 23;11:561298. doi: 10.3389/fmicb.2020.561298 (PMC7644646; doi:10.3389/fmicb.2020.561298)

Supplementary material

Inhibitory effect of morin against *Candida albicans* pathogenicity and virulence factor production: an *in vitro* and *in vivo* approaches

Gurusamy Abirami[1], Rajaiah Alexpandi[1], Ravindran Durgadevi[1], Arunachalam Kannappan[1,2], Arumugam Veera Ravi[1]*

[1]Department of Biotechnology, School of Biological Sciences, Alagappa University, Karaikudi 630003, Tamil Nadu, India.

[2]Department of Food Science and Technology, School of Agriculture and Biology, Shanghai Jiao Tong University, Shanghai 200240, China.

Corresponding Author:

Dr. Arumugam Veera Ravi

Professor

Department of Biotechnology

Alagappa University

Karaikudi-630003

India.

Email: aveeraravi@rediffmail.com

Supplementary Figures

Figure S1 **(A)** Enumeration of filamentous cells in the presence and absence of morin at its MBIC. **(B)** Light microscopic analysis of single spot filamentous cells in the presence and absence of morin at its MBIC were represented. Data are presented as means ± SD. * indicates the statistical significance ($p < 0.05$)

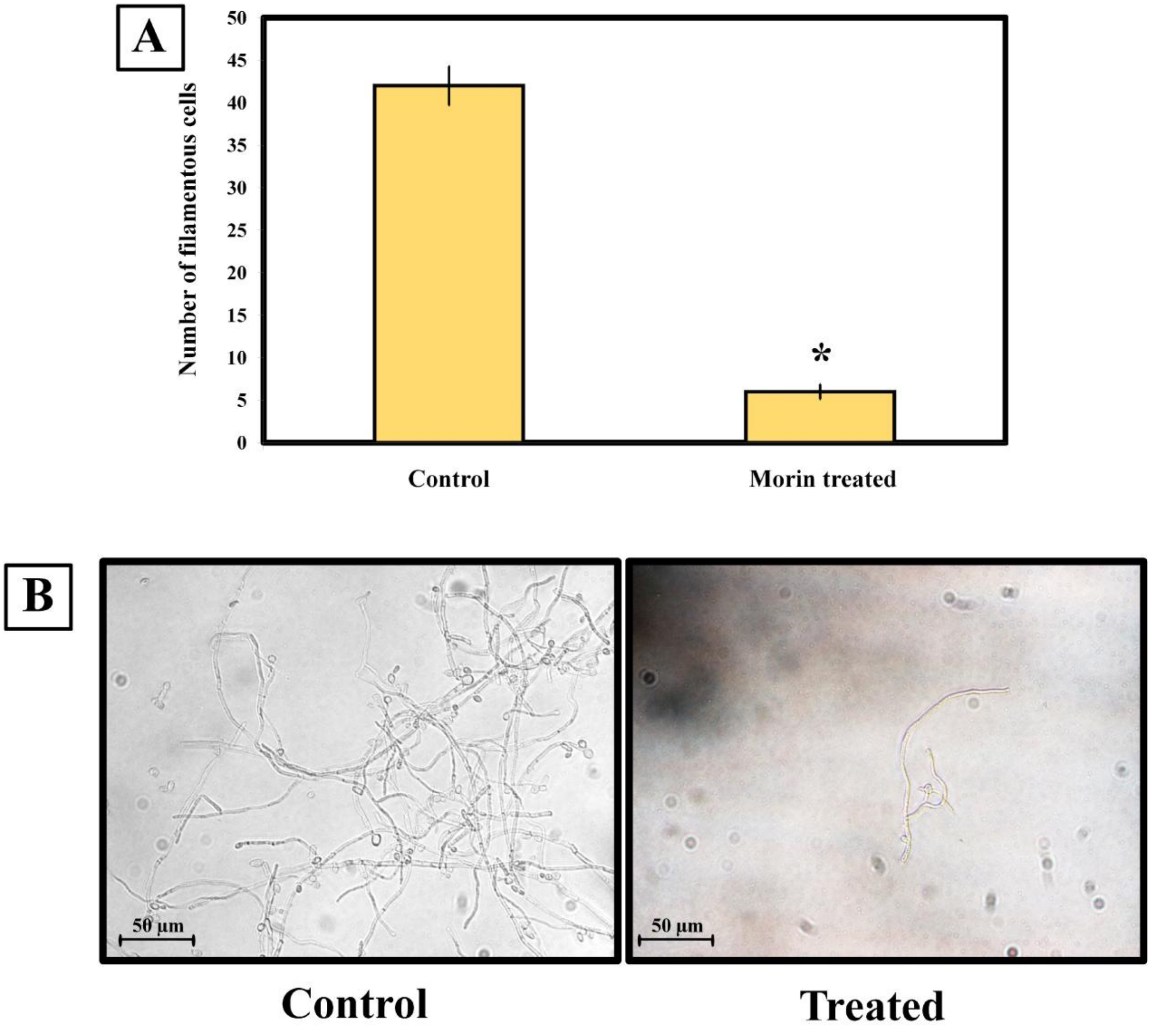

Figure S2 **(A)** Inhibitory effect of morin on *C. albicans* mature biofilm production. **(B)** Light microscopic images showing the effective non-inhibitory activity of morin at MBIC on preformed biofilm of *C. albicans*.

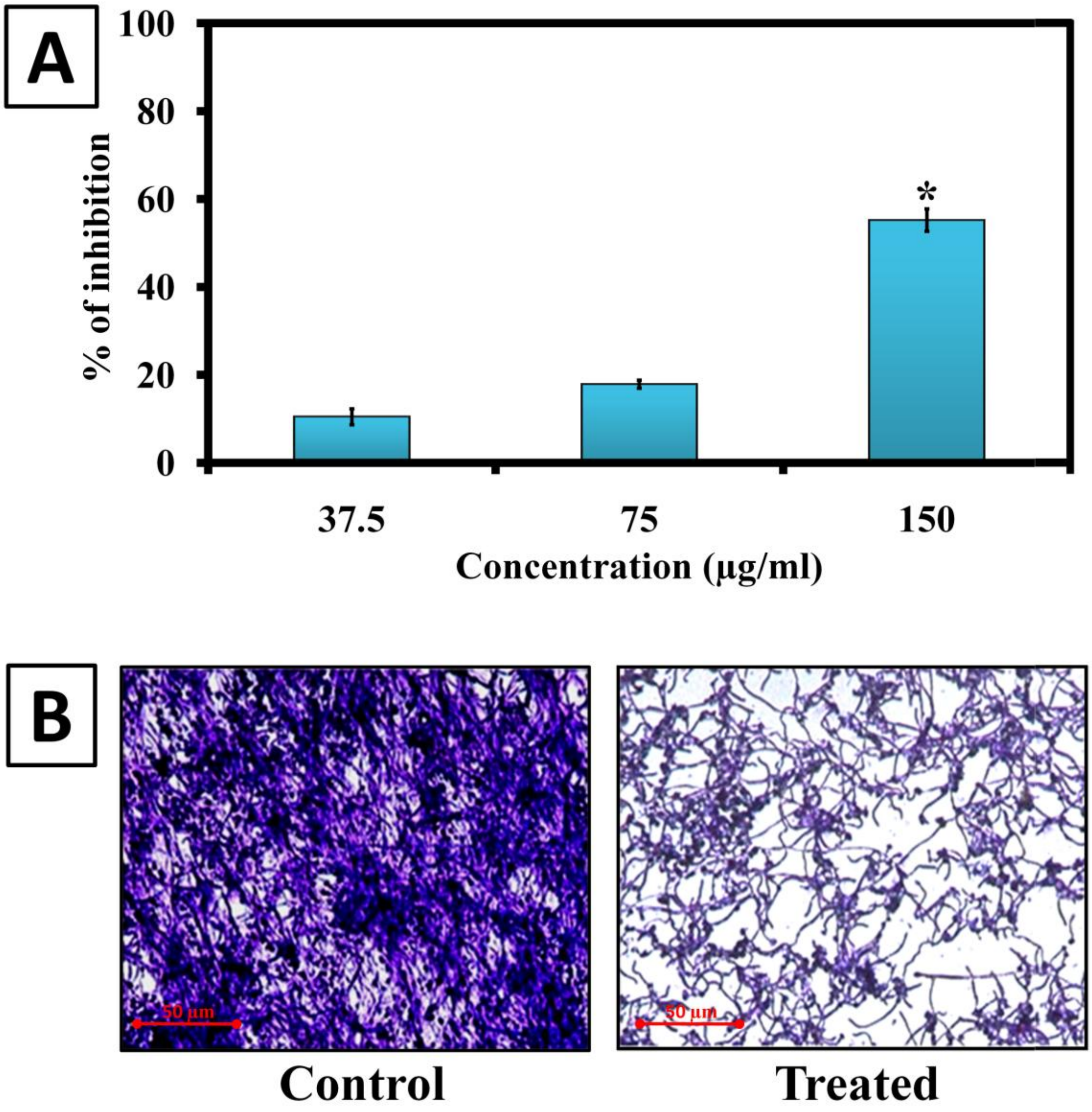

Figure S3 Photographs illustates the clinical symptoms of *C. albicans* infection in zebrafish in the presence and absence of morin treatment. In *C. albicans* (CA) infected group, the black arrows represent the haemorrhaging at visceral organs of zebrafish such as (a) gills (b) gastrointestinal tract (c) urinary tract upon *C. albicans* infection.

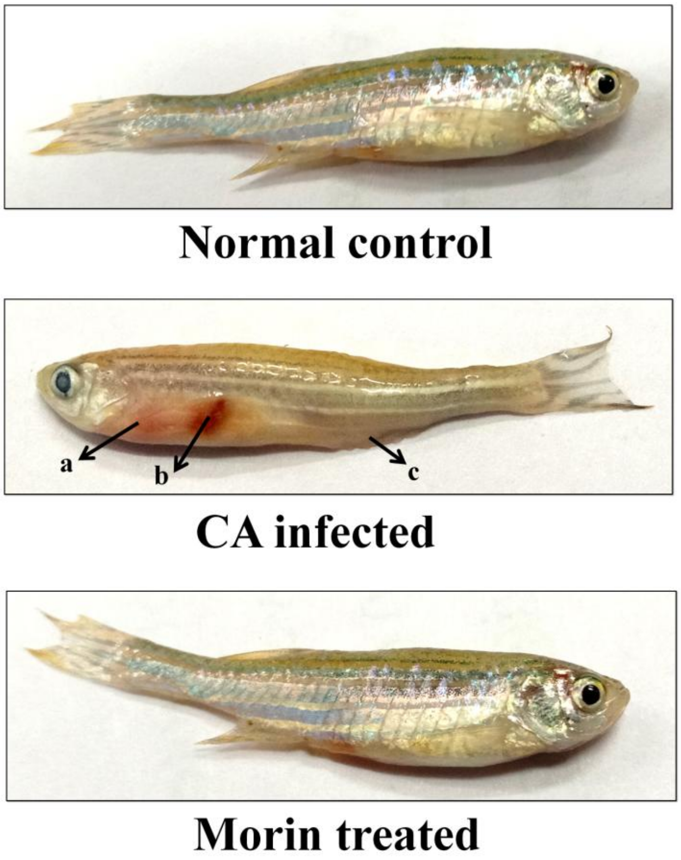

Figure S4 Effect of morin on *in vivo* colonization of *C. albicans* in zebrafish. The reduction number of *C. albicans* colonization on zebrafish was assessed by CFU assay.

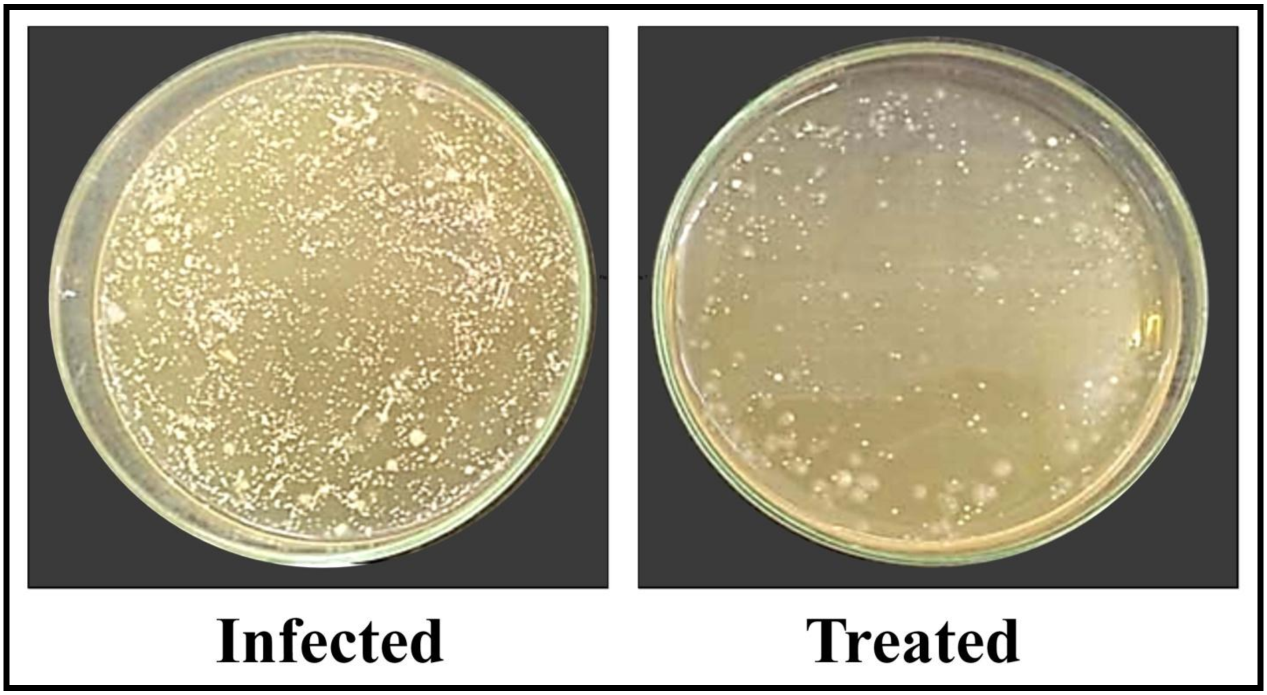

Supplement: Supplementary Figure 1 — (A) Enumeration of filamentous cells in the presence and absence of morin at its MBIC. Data are presented as means ± SD. ∗ indicates the statistical significance (p < 0.05). (B) Light microscopic analysis of single spot filamentous cells in the presence and absence of morin at its MBIC were represented. [file Data_Sheet_1.PDF]
